# Cooperative role of endogenous leucotrienes and platelet-activating factor in ischaemia–reperfusion-mediated tissue injury

**DOI:** 10.1111/jcmm.12118

**Published:** 2013-11-01

**Authors:** Claudia S Bitencourt, Valérie L Bessi, David N Huynh, Liliane Ménard, Julie S Lefebvre, Tania Lévesque, Leila Hamdan, Fanny Sohouhenou, Lucia H Faccioli, Pierre Borgeat, Sylvie Marleau

**Affiliations:** a Department of Clinical Analysis, Toxicology and Bromatology, Faculty of Pharmaceutical Sciences of Ribeirão Preto, University of São PauloRibeirão Preto, São Paulo, Brazil; b Faculty of Pharmacy, Université de MontréalMontréal, QC, Canada; c Rheumatology and Immunology Research Center, CHUQ Research Center and Université LavalLaval, QC, Canada

**Keywords:** Ischaemia/reperfusion, cell trafficking, dermal inflammation, lipid mediator, polymorphonuclear neutrophil, myocardial infarction, leucotrienes, platelet-activating factor

## Abstract

Insufficient oxygen delivery to organs leads to tissue dysfunction and cell death. Reperfusion, although vital to organ survival, initiates an inflammatory response that may both aggravate local tissue injury and elicit remote organ damage. Polymorphonuclear neutrophil (PMN) trafficking to remote organs following ischaemia/reperfusion (I/R) is associated with the release of lipid mediators, including leucotriene (LT) B_4_, cysteinyl-LTs (CysLTs) and platelet-activating factor (PAF). Yet, their potentially cooperative role in regulating I/R-mediated inflammation has not been thoroughly assessed. The present study aimed to determine the cooperative role of lipid mediators in regulating PMN migration, tissue oedema and injury using selective receptor antagonists in selected models of I/R and dermal inflammation. Our results show that rabbits, pre-treated orally with BIIL 284 and/or WEB 2086 and MK-0571, were protected from remote tissue injury following I/R or dermal inflammation in an additive or synergistic manner when the animals were pre-treated with two drugs concomitantly. The functional selectivity of the antagonists towards their respective agonists was assessed *in vitro*, showing that neither BIIL 284 nor WEB 2086 prevented the inflammatory response to IL-8, C5a and zymosan-activated plasma stimulation. However, these agonists elicited LTB_4_ biosynthesis in isolated rabbit PMNs. Similarly, a cardioprotective effect of PAF and LTB_4_ receptor antagonists was shown following myocardial I/R in mice. Taken together, these results underscore the intricate involvement of LTB_4_ and PAF in each other’s responses and provide further evidence that targeting both LTs and PAF receptors provides a much stronger anti-inflammatory effect, regulating PMN migration and oedema formation.

## Introduction

Ischaemia–reperfusion (I/R) injury is the consequence of insufficient oxygen delivery to an organ followed by effective tissue reperfusion. This initiates an inflammatory response that may both aggravate local injury and elicit remote organ dysfunction. Ischaemia–reperfusion injury may occur in the context of acute arterial occlusion leading to myocardial infarction and stroke, limb ischaemia, or following surgical procedures 1,2. Along that line, concomitant arterial and venous occlusion of hind limbs is characterized by local and distant organ injury that is exacerbated following reperfusion of ischaemic limbs, with the lungs as the most vulnerable target organ 4. During the ischaemic period, there is inhibition of oxidative phosphorylation that is accompanied by various changes, such as lactate accumulation and intracellular acidification, conducive to loss of ion homeostasis and calcium overload 5. The elevation in cytosolic Ca^2+^ is further accelerated after reperfusion and promotes membrane phospholipid breakdown, the formation of cytotoxic products and phospholipase A_2_ activation 6. In addition, reintroduction of molecular oxygen with blood flow restoration increases polymorphonuclear neutrophil (PMN) adhesion to the microvascular endothelium, activation and release of reactive oxygen species (ROS) and vasoactive and inflammatory mediators, promoting damage to local and peripheral tissues 7. In agreement, early studies using first-generation receptor antagonists and enzyme inhibitors supported a role for lipid mediators, including platelet-activating factor (PAF) 8, leucotriene (LT) B_4_ 9–10 and prostanoids 11, in mediating post-ischaemic vasoactive and/or leucocyte adhesion events. The relative importance and potentially cooperative role of these mediators in the context of I/R injury, however, is yet to be clearly demonstrated.

In particular, LTB_4_ has long been recognized as a potent chemotactic lipid mediator signalling mainly through high (BLT1)- and low (BLT2)- affinity receptors in mediating leucocyte activation and recruitment in acute inflammation and host defence 12–13. Leucotriene B_4_ has also been ascribed a pathogenic role in chronic inflammatory diseases, including arthritis, inflammatory bowel disease, sepsis, psoriasis and asthma, which led to the development of selective and potent receptor antagonists as a novel pharmacological approach for the treatment of inflammatory diseases 14. In acute inflammation, such as that observed following limb I/R, *de novo* LTB_4_ generation has been associated with PMN activation and distant tissue injury 9. In addition to LTB_4_ biosynthesis, the adhesion of activated PMNs to microvascular endothelial cells also leads to an increased biosynthesis of endothelial cysteinyl-containing leucotrienes (CysLTs) through transcellular metabolism of LTA_4_ 15. In this context, CysLTs may contribute to increasing vascular permeability and tissue oedema 16, also triggering PAF biosynthesis 17. Similarly, PAF is concomitantly biosynthesized with eicosanoids within activated leukocytes 18 and endothelial cells 19 and has been ascribed an important early role in I/R injury 20. Both PAF and LTB_4_ may act in an autocrine/paracrine 21 and intracrine 22 fashion to enhance intracellular arachidonic acid availability and LT biosynthesis. The interplay between these mediators is also illustrated by the role of BLT1 signalling in PAF-induced degranulation and chemotaxis of PMNs 23.

Despite evidence for an important role of lipid mediators in both acute and chronic inflammatory responses, targeting a single mediator has provided limited therapeutic benefit. The present study aimed to elucidate whether LTs and PAF act in a cooperative manner to regulate plasma extravasation and PMN trafficking to inflammatory sites in experimental models of I/R using potent and selective BLT1/BLT2 and PAF receptor (PAFR) antagonists. Hind limb and myocardial I/R models in two species underscored the cooperative role of arachidonic acid-derived mediators, whereas skin bioassay allowed delineation of their relative contribution in PMN accumulation and tissue injury.

## Materials and methods

### Animals

All experimental procedures were approved by the Institutional Animal Ethics Committee of the Université de Montréal, in accordance with the Canadian Council on Animal Care guidelines for use of experimental animals. Male New Zealand rabbits (2.5–3 kg) were purchased from Charles River (St-Constant, QC, Canada). They were housed in individual cages with free access to food (Purina pellets) and water *ad libitum* for ∼5 days prior to the terminal experiment. Mice deficient in PAF receptor (PAFR^−/−^) and their C57BL/6 control littermates were obtained from Dr. S. Ishii and T. Shimizu, University of Tokyo, and bred in-house.

### Materials

BIIL 284 and its active metabolite BIIL 260, as potent BLT1 and BLT2 receptor antagonists, and WEB 2086, a selective PAF receptor antagonist, were kindly provided by Dr. F. Birke (Boehringer Ingelheim Pharma GmbH & Co., Biberach/Riss, Germany). 5(*S*),12(*R*)-Dihydroxy-6,14-*cis*-8,10-*trans*-eicosatetraenoic acid (LTB_4_) was purchased at Cayman Chemicals (Ann Harbor, MI, USA) and MK-0571, a LTD_4_ (CysLT1) receptor antagonist, was generously provided by Merck Sharp & Dohme Research laboratories, Kirkland, QC, Canada. *N*-(2-dimethylaminoethyl)-*N*-(3-pyridinylmethyl) (4-(2,4,6-triisopropyl-phenyl)thiazol-2-yl)amine (SR 27417) was kindly provided by Dr. J.-M. Herbert (Sanofi-Aventis, Toulouse, France). Bovine serum albumin (BSA; very low endotoxin), dimethylsulfoxide, hexadecyltrimethylammonium bromide (HTAB), hydrogen peroxide (H_2_O_2_), luminol (5-amino-2,3-dihydro-1,4-phtalazinedione), *N*-formyl-methionyl-leucyl-phenylalanine (fMLP), PAF, prostaglandin E_2_ (PGE_2_), 3,3′, 5,5′-tetramethylbenzidine (TMB) and zymosan A were purchased from Sigma-Aldrich (St-Louis, MO, USA). Interleukin (IL)-8 and tumour necrosis factor-α (TNF-α) were purchased from PeproTech Inc. (Rocky Hill, NJ, USA). Pyrrophenone was obtained from Shionogi Research Laboratories (Osaka, Japan). Modified Hank’s balanced salt solution (HBSS), hydroxyethyl piperazineethanesulfonic acid (HEPES) buffer and DMEM were from Gibco Life Technologies (Grand Island, NY, USA). Ketamine was bought from Vetrepharm-Canada Inc. (Belleville, ON, Canada) and xylazine from Bimeda-MTC Animal Health Inc. (Cambridge, ON, Canada). Acepromazine was purchased from Ayerst Veterinary Laboratories (Guelph, ON, Canada) and isoflurane USP was obtained from Pharmaceutical Partners of Canada Inc. (Richmond, ON, Canada). Pentobarbital (Euthansol) was obtained from Schering-Plough Canada Inc. (Pointe-Claire, QC, Canada). All other solutions for parental administration were purchased from Baxter Corporation Laboratories (Toronto, ON, Canada).

### Hind limb ischaemia–reperfusion

The rabbits were pre-treated orally with either BIIL 284 (0.01 mg/kg), WEB 2086 (10 mg/kg) or both 2 hrs before subjecting them to bilateral ischaemia of the lower limbs. Briefly, sedation was induced by acepromazine (0.5 mg/kg), and anaesthesia was induced and maintained by inhaled anaesthetic mixture of oxygen (2 l/min.) and 3–5% isoflurane administered through a face mask. The animals were maintained on heating pads throughout the experimental protocol. Transient ligation of the femoral arteries, veins and collateral vessels was performed by cutting soft-tissue and bony structures, exclusive of femoral vessels around the thighs and by clamping all the vessels *in situ* with a microvascular clip. For sham-operated controls, the same surgical procedure was performed without clamping of the vessels. After a 2-hour period of ischaemia, the clips were removed to allow reperfusion for 4 hrs. Blood samples (2 ml) were taken at 0, 2 and 6 hrs from the central artery of the ear for total and differential leucocyte counts and for chemiluminescence assay. Rabbits were killed with an overdose of pentobarbital. The heart was flushed with 180 ml of 0.9% NaCl containing 10 UI heparin and tissue aliquots of lungs, intestine (jejunum) and liver (left lobe) were snap-frozen in liquid nitrogen and kept at −80°C until assayed for myeloperoxidase (MPO) activity.

### Whole blood chemiluminescence

Luminol-enhanced whole blood chemiluminescence was studied using opsonized zymosan (OpZ, 1 mg/ml) as a stimulus. Briefly, heparinized blood was collected and processed immediately. Blood was diluted (1/10) in DMEM containing 50 mM HEPES and 4 μM luminol and incubated under continuous stirring for 5 min. at 37°C before stimulation. The chemiluminescence signals were recorded using a computer-assisted luminometer (model 500; Chronolog Corp, Havertown, PA, USA). Chemiluminescence intensities were measured as the peak amplitude in arbitrary units.

### Dermal inflammation

On the day of the experiment, WEB 2086 was diluted in 0.5% carboxymethylcellulose (CMC) suspension and BIIL 284 was diluted in sterile water. Both drugs were administered orally (1 ml/kg) 2 hrs before intradermal (i.d.) injections. The rabbits were sedated with acepromazine (0.5 mg/kg) and anaesthetized with a solution of ketamine/xylazine (35:5) (0.4 ml/kg) and the dorsal area was shaved. The agonists under study, including LTB_4_ (500 pmol/site), PAF (100 pmol/site), TNF-α (10 pmol/site), IL-8 (10 pmol/site) and zymosan-activated plasma (ZAP, 1%), were diluted in HBSS containing 0.1% BSA (HBSS/0.1% BSA). Each agonist was injected i.d. (50 μl by site) in quadruplicate, according to a predetermined site plan. The accumulation of PMNs into skin sites over a 2-hour period was assessed by measuring MPO activity. Briefly, the rabbits were killed with an overdose of pentobarbital, the dorsal skin was cleansed of blood and fat and biopsies (11 mm diameter) were punched out and frozen at −80°C until assayed. Blood samples (2 ml) were taken from the central artery of the ear for total and differential leucocyte counts at 0, 60 and 120 min. following i.d. injections.

### Vascular permeability and oedema

In additional experiments, the rabbits received an i.v. injection of ^125^I-BSA (1 μCi/kg) 30 min. before killing. Five minutes later, agonists were injected i.d. (50 μl/site), including LTD_4_ (1000 pmol/site) and LTC_4_ (160 pmol/site), to assess vascular permeability. Biopsies and plasma were counted in a gamma counter (1277 GammaMaster, PerkinElmer Life Sciences, Boston, MA, USA) and oedema was quantified as μl plasma extravasated = CPM (biopsy)/CPM (1 μl plasma). In I/R experiments, tissue oedema was assessed by calculating the blood-free wet-to-dry weight ratio. Briefly, anaesthetized rabbits were killed by an overdose of pentobarbital, the lungs, liver and intestine were flushed *in situ*, removed, blotted to remove excess fluid and the wet weights were recorded. Tissues were then placed in an incubator at 60°C for 5 days, and the dry weights were recorded 24.

### MPO assay

Tissue MPO activity was assayed as previously described 25, with some modifications. Briefly, tissues were homogenized in 1 ml of PBS and the pellets, as well as skin biopsies, were homogenized in 1 ml acetate buffer (100 mM), pH 6.0, containing 0.5% HTAB and 20 mM ethylenediaminetetraacetic acid (EDTA). Tissue homogenates, except skin biopsies, were heated to 65°C for 120 min. in a water bath. The homogenates were subjected to three freeze–thaw cycles and then centrifuged at 2000 *g* for 10 min. Myeloperoxidase (freed from PMN granules) was assayed by incubating supernatants with 3.2 mM TMB and 0.3 mM H_2_O_2_ for 5 min. at 37°C. The reaction was stopped by the addition of 100 μl of 0.2 M sodium acetate (pH 3.0). Polymorphonuclear neutrophil calibration curves were prepared using rabbit peritoneal PMNs following i.p. glycogen stimulation (0.2% glycogen, 4 hrs accumulation). Polymorphonuclear neutrophil numbers were calculated from the standard curves and expressed as PMNs per site or per g of tissue.

### Rabbit PMN isolation and *in vitro* stimulation and chemotaxis assay

Peripheral rabbit PMNs were isolated on Percoll gradient, as described before 13. Freshly isolated PMNs (5 × 10^6^/ml) were suspended in HBSS containing 10% foetal bovine serum and 1.6 mM CaCl_2_ and incubated with 5 μg/ml calcein-AM (Molecular Probes, Eugene, OR, USA) at 37°C for 30 min. in the dark with gentle agitation. The cells were then washed and resuspended in HBSS/FBS/Ca^2+^. Polycarbonate filters with 3 μm pore size (96-well ChemoTx disposable chemotaxis system from Neuro Probe, Gaithersburg, MD, USA) were used for PMN chemotaxis. Cell suspension (25 μl) at 3 × 10^6^ PMNs/ml was placed on the filter and PMNs were allowed to migrate for 1 hr at 37°C and 5% CO_2_ in the dark. Non-migrating cells were gently removed, and cell migration was measured with a microplate fluorescence reader (FL600; BioTek Instruments, Winooski, VT, USA) using 485 nm excitation and 530 nm emission wavelengths. Fluorescence corresponding to a known number of cells was obtained and the results expressed as per cent migrating cells. Isolated PMNs (3–5 × 10^6^) were stimulated with various soluble agonists in HBSS/Ca^2+^ for 15 min., then the cells were centrifuged, proteins denatured in methanol and the samples were kept for analysis by LC-MS as described previously 26.

### Myocardial ischaemia–reperfusion in mice

Myocardial I/R was performed according to Bessi *et al*. 27. Mice were pre-treated orally with BIIL 284 (0.1 mg/kg), SR 27417 (0.3 mg/kg) or both drugs 2 hrs before subjecting them to myocardial I/R. Briefly, mice undergoing a 30-minute ischaemia and 48-hour reperfusion received an intraperitoneal injection of buprenorphine (0.05 mg/kg), and were then intubated orally and connected to a Minivent mouse ventilator (Harvard Apparatus Canada, Saint-Laurent, QC, Canada). Mice were anaesthetized with 3% isoflurane mixed with 100% oxygen. The adequacy of anaesthesia was monitored using loss of pedal withdrawal reflex. Anaesthesia was maintained with 1.5–2% isoflurane and body temperature was maintained using a heating pad. A left lateral incision (third intercostal space) was performed to provide exposure of the left anterior descending (LAD) coronary artery. An 8-0 nylon suture was placed 1 mm distal to the edge of the atrial appendage and tied over a piece of tubing (Silastic® brand; 0.30 × 0.64 mm). Ischaemia was confirmed by visual pallor and reperfusion by reversal of this effect. To prevent cardiac arrhythmia, two intraperitoneal doses of lidocaine (6 mg/kg) were given, one prior to occlusion and a second prior to reperfusion. The chest cavity and skin were closed separately using a 6-0 silk suture and the animals were allowed to recover for 48 hrs.

### Myocardial infarct size and leucocyte infiltration

Before killing, mice underwent surgical preparation and a suture was re-tied at the site of the original ligation. Injection of 1 ml Evans blue dye solution (1%) was used to delineate the heart non-ischaemic area and 2,3,5-triphenyltetrazolium chloride was used to determine the non-infarcted ischaemic area. The left ventricle (LV) area and the infarcted area (IA) were determined by computerized planimetry using Adobe CS3 Photoshop software (Ottawa, ON, Canada), as detailed previously 27. For the assessment of myocardial leucocyte infiltration, anaesthetized mice were perfusion-fixed *in situ* by infusion with 10% neutral-buffered formalin at a constant rate of 1.5 ml/min. for 7 min. Haematoxylin and eosin staining was performed at the Histology core platform, Institut de recherche en immunologie et cancérologie, Université de Montréal.

### Statistical analysis

All results are expressed as mean ± SEM, and statistical comparisons were carried out by unpaired *T*-test or a one-way anova followed by Student-Newman-Keuls pair-wise multiple comparisons using GraphPad Prism Version 4.0 (San Diego, CA, USA), where appropriate. Differences were considered significant at *P* < 0.05.

## Results

### Cooperative role of LTB_4_ and PAF in leucocyte recruitment and plasma extravasation to remote organs following reperfusion of ischaemic lower limbs in rabbits

**Figure 1:**
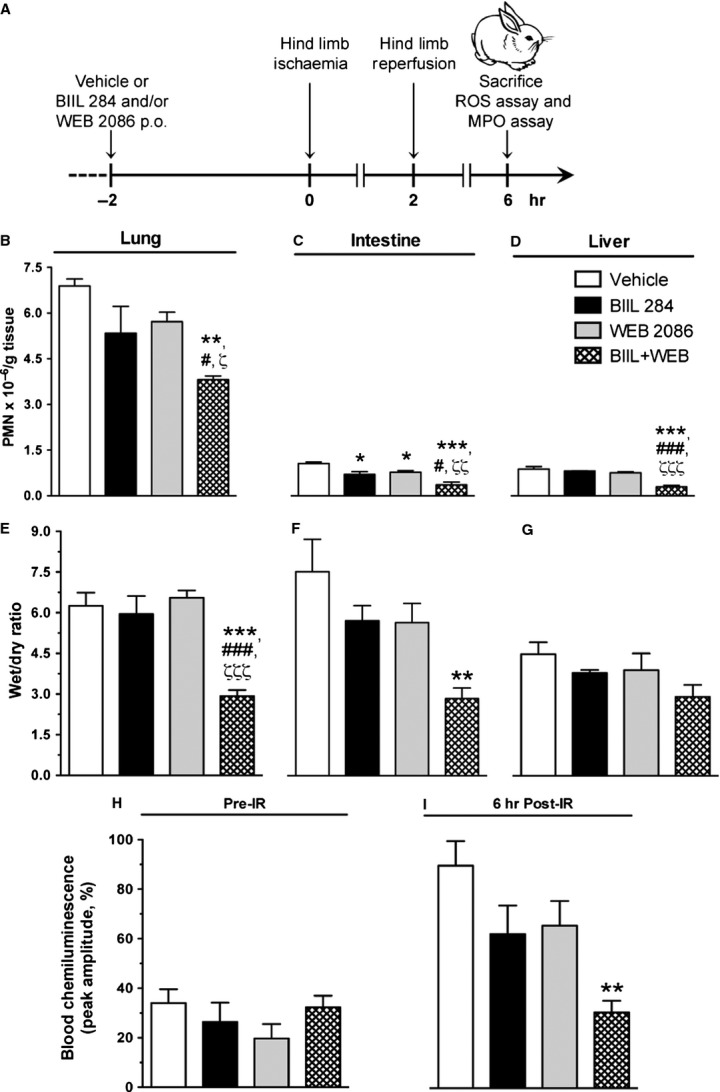
Effect of BIIL 284 and WEB 2086 on polymorphonuclear neutrophil (PMN) recruitment and oedema to remote organs and whole blood chemiluminescence following hind limb I/R in rabbits. (A) Schematic representation of the experimental protocol involving the oral administration of BIIL 284 (0.01 mg/kg), WEB 2086 (10 mg/kg) or both drugs 2 hrs before subjecting rabbits to bilateral ischaemia of the lower limbs. The effect of single or combined drug administration on PMN recruitment to (B) lungs (left lobe), (C) intestine (jejunum) and (D) liver as assessed by myeloperoxidase assay. The effect of drugs on tissue oedema of (E) lungs (right lobe), (F) intestine and (G) liver as assessed by determining the wet/dry ratio. The effect of drug treatment on reactive oxygen species generation in whole blood stimulated by OpZ (H) before, and 6 hrs after I/R of lower limbs, as assessed by blood chemiluminescence. Data are the mean ± SEM obtained from four rabbits. *P < 0.05, **P < 0.01, ***P < 0.001 *versus* water; ^#^*P* < 0.05, ^###^*P* < 0.001 *versus* BIIL 284; ^ζ^*P* < 0.05, ^ζζ^*P* < 0.01, ^ζζζ^*P* < 0.001 *versus* WEB 2086.

The first series of experiments aimed at determining the putative collaborative role of LTB_4_ and PAF in regulating PMN recruitment to remote organ following acute I/R of lower limbs. To do so, rabbits were pre-treated with the LTB_4_ receptor antagonist BIIL 284 and/or the PAF receptor antagonist WEB 2086 orally 2 hrs before being subjected to bilateral ischaemia of lower limbs for a 2-hour period, followed by 4 hrs of reperfusion (Fig. 1A). Polymorphonuclear neutrophil recruitment to the lungs was ∼sevenfold higher in lungs than in intestine or liver (Fig. 1 B–D). While either BIIL 284 or WEB 2086, administered alone, did not significantly attenuate PMN recruitment to the lungs, the concomitant administration of these drugs had an additive inhibitory effect and reduced PMN accumulation by 45% (*P* < 0.01) compared to vehicle (Fig. 1B). An additive inhibitory effect of dual PAF and LTB_4_ receptor antagonist administration was also observed on PMN recruitment to the intestine (Fig. 1C), while a synergistic effect of a combination of the two drugs was observed in the liver (Fig. 1D). In a similar manner, lung and intestinal oedema was reduced significantly only in animals having received both treatments (Fig. 1E and F) with the same tendency for the liver (Fig. 1G). Further supporting a cooperative role of LTB_4_ and PAF in the systemic inflammatory response triggered by I/R of the lower limbs is the finding that the production of ROS in whole blood stimulated with OpZ was significantly reduced (66% lower than control, *P* < 0.01) only when the rabbits were pre-treated with both BIIL 284 and WEB 2086 (Fig. 1H and I).

### Cooperative role of LTs and PAF in dermal inflammation elicited by a variety of agonists in a rabbit bioassay model

**Figure 2:**
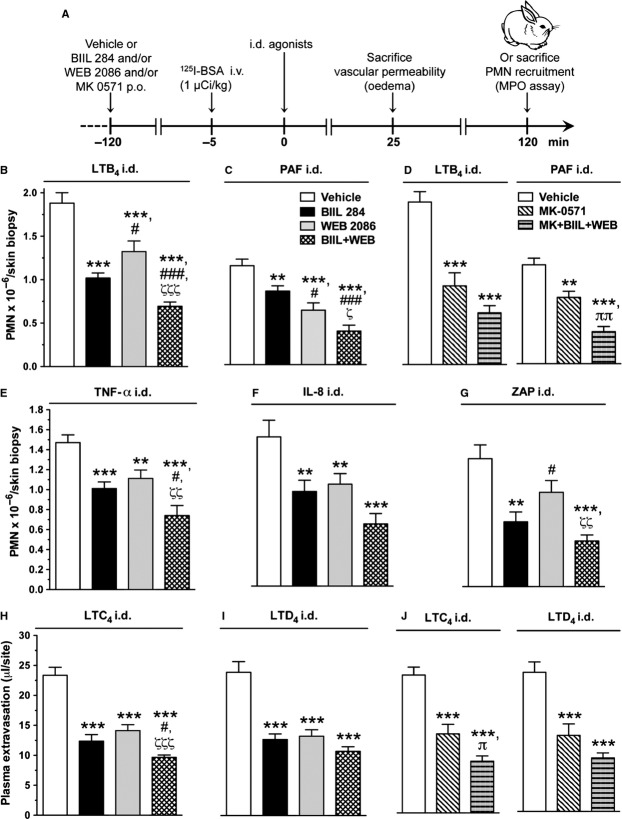
Effect of BIIL 284 and WEB 2086 on polymorphonuclear neutrophil (PMN) recruitment and oedema to dermal inflammatory sites. (A) Schematic representation of the experimental protocol allowing the assessment of the effect of pharmacological treatment with BIIL 284 (0.01 mg/kg), WEB 2086 (10 mg/kg), MK-0571 (10 mg/kg) or a combination of these drugs on plasma extravasation and PMN trafficking to inflammatory sites elicited by the injection of various soluble agonists injected i.d. The agonists under investigation included (B) LTB_4_ (500 pmol/site), (C) PAF (100 pmol/site, containing 250 pmol PGE_2_) and (D) LTB_4_ or PAF, (E) TNF-α (10 pmol/site), (F) IL-8 (10 pmol/site) and (G) ZAP (1%). PMN accumulation was assessed in homogenized skin biopsies using the myeloperoxidase assay. Plasma extravasation was assessed following the i.d. injections of (H) LTC_4_ (160 pmol/site) and (I) LTD_4_ (1 nmol/site) and (J) LTC_4_ or LTD_4_, as assessed following the injection of ^125^I-BSA (1 μCi/kg) 30 min. prior to killing. Data are the mean ± SEM obtained from 16 biopsies. **P < 0.01, ***P < 0.001 *versus* water; ^#^*P* < 0.05, ^###^*P* < 0.001 *versus* BIIL 284; ^ζ^*P* < 0.05, ^ζζ^*P* < 0.01, ^ζζζ^*P* < 0.001 *versus* WEB 2086; ^π^*P* < 0.05, ^ππ^*P* < 0.01 *versus* MK-0571.

To better evaluate the relative role of lipid mediators, as well as the extent of the cooperative role of LTs and PAF in inflammatory responses, another series of experiments was undertaken using a dermal inflammation model in rabbits. As detailed in the experimental scheme presented in Figure 2A, the role of endogenously produced LTB_4_, PAF and CysLTs in oedema and PMN recruitment to the skin in response to various agonists was investigated using their respective receptor antagonists BIIL 284, WEB 2086 and MK-0571. The results show that dual PAF and LTB_4_ receptor blockade exerted an additive inhibitory effect (*P* < 0.001) on PMN recruitment to dermal skin sites after the injection of various agonists, compared with the administration of either drug alone (Fig. 2B–G). The results also show that the doses of BIIL 284 or WEB 2086 used in the current studies inhibited PMN recruitment elicited by their respective agonist by approximately 45% (*P* < 0.001). Interestingly, BIIL 284 hindered PAF-induced PMN accumulation and WEB 2086 prevented LTB_4_-induced PMN trafficking by ∼25–30% (*P* < 0.01–0.001; Fig. 2B and C), underlining the intricate involvement of LTB_4_ and PAF in each other’s responses. Furthermore, each drug was able to partially block PMN accumulation to chemically unrelated inflammatory mediators, including the cytokine TNF-α, the chemokine IL-8 and to ZAP, a source of activated complement peptide C5a des-arginine (*P* < 0.05–0.001; Fig. 2E–G). In addition, the combination of both LTB_4_ and PAF receptor antagonists had an additive inhibitory effect, reducing PMN recruitment by up to 50–64% (*P* < 0.001; Fig. 2B–G). As mentioned previously, as CysLT biosynthesis is tightly linked to that of LTB_4_ and PAF, it was of interest to delineate the potential role of CysLTs in regulating PMN trafficking and plasma extravasation. Both LTB_4_- and PAF-induced PMN accumulation to skin sites was partially blocked by the LTD_4_ receptor antagonist (MK-0571), and the addition of MK-0571 to BIIL 284 and WEB 2086 treatment regimen further reduced PMN trafficking (Fig. 2D). A single administration of BIIL 284 and WEB 2086 impeded LTC_4_- and LTD_4_-induced skin oedema by ∼40% (*P* < 0.001), and the effect of the combined drug treatment was slightly enhanced (Fig. 2H and I). In a similar manner, MK-0571 reduced the effect of CysLTs in enhancing vascular permeability, and adding BIIL 284 and WEB 2086 to the treatment regimen further had modest additional effect in preventing skin oedema formation (Fig. 2J).

### LTs and PAF receptor antagonists as selective inhibitors of rabbit PMN chemotaxis *in vitro*

**Figure 3:**
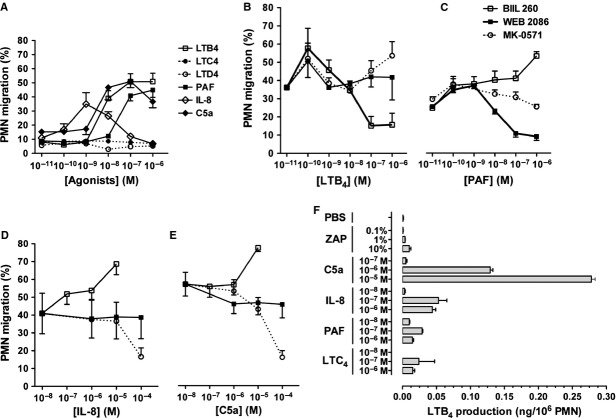
The effect of BIIL 260, WEB 2086 and MK-0571 on rabbit polymorphonuclear neutrophil (PMN) chemotaxis through polycarbonate filters. PMNs were labelled with calcein-AM and (A) Concentration-response curves in response to chemotactic agonists were assessed as described in Material and methods. Concentration-inhibition curves of PMN chemotaxis by BIIL 260, WEB 2086 and MK-0571 on (B) LTB_4_-, (C) PAF-, (D) IL-8- and (E) C5a-elicited PMN chemotaxis. The experiments were performed in the presence of 100 nM pyrrophenone. (F) Concentration dependence of LTB_4_ generation following stimulation of isolated rabbit PMNs with soluble agonists. Data are the mean of three independent experiments.

Although BIIL 284, WEB 2086 and MK-0571 are well-recognized receptor antagonists for LTB_4_, PAF and LTD_4_, respectively, their efficacy and selectivity on rabbit receptors have not been documented. We therefore investigated the functional selectivity of these antagonists against their respective agonists in a chemotaxis assay using freshly isolated PMNs from rabbit peripheral blood. All agonists, but LTC_4_ and LTD_4_, which are also known for their lack of chemotactic activity for human PMNs 28, stimulated the chemotaxis of rabbit PMNs through polycarbonate filters (Fig. 3A). The results show that BIIL 284 and WEB 2086 dose-dependently inhibited LTB_4_ and PAF-induced migration, respectively (Fig. 3B and C), but not that of IL-8, C5a (Fig. 3D and E) and ZAP (data not shown). However, an apparent increase in PMN chemotaxis in response to the soluble agonists IL-8 and C5a was observed in the presence of the BLT1/2 receptor antagonist, but not with the PAF receptor antagonist, which may indicate a role for PAF in mediating, in part, PMN trafficking in response to elevated (>10^−6^M) concentration of agonists. Our results also show that all agonists and, in particular, C5a and IL-8 elicited LTB_4_ biosynthesis by isolated rabbit PMNs (Fig. 3F).

### Cooperative role of LTB_4_ and PAF in mediating tissue injury following transient myocardial ischaemia in mice

**Figure 4:**
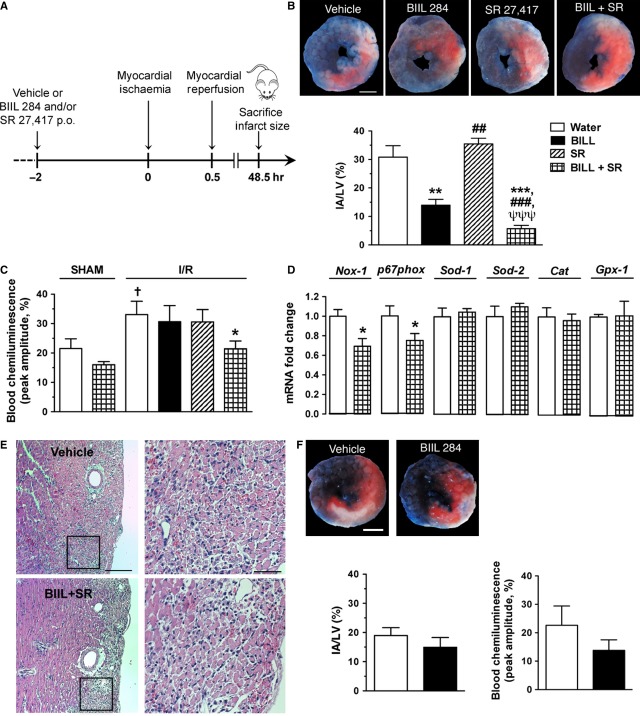
BIIL 284 and SR 27417 reduced infarcted areas following myocardial I/R in wild-type (WT) mice. (A) Schematic representation of the experimental protocol involving the oral administration of BIIL 284 (0.1 mg/kg), SR 27417 (0.3 mg/kg) or both drugs 2 hrs before subjecting them to transient myocardial ischaemia following the occlusion of the left anterior descending coronary artery. (B) Representative photomicrographs of the mid-ventricular myocardium showing the area at risk [red staining including infarcted (white) tissue] and non-ischaemic (blue staining) section of left ventricle and a bar graph showing the effect of drug pre-treatment on infarct area (*n* = 5–7 WT mice per group); scale bar = 1 mm. (C) The effect of drug treatment on reactive oxygen species generation in whole blood stimulated by OpZ at 48 hrs following myocardial I/R, as assessed by blood chemiluminescence (*n* = 4 sham-operated, and *n* = 6–12 I/R). (D) The effect of drug treatment on gene expression at 48 hrs following myocardial I/R, as assessed by qPCR of left ventricule (LV) homogenates (*n* = 7–8). (E) Representative photomicrographs of mid-ventricular LV sections of a mouse treated with vehicle or BIIL 284 and SR 27417; scale bar = 200 and 20 μm for left (100 × ) and right (400 × ) images respectively. (F) Representative photomicrographs and bar graphs showing the effect of BIIL 284 pre-treatment on infarct size in PAFR^−/−^ mice (*n* = 4–5 mice per group). Data are mean ± SEM. **P < 0.01, ***P < 0.001 *versus* water; ^##^*P* < 0.01, ^###^*P* < 0.001 *versus* BIIL 284; ^ψψψ^*P* < 0.001 *versus* SR 27417; †*P* < 0.05 *versus* sham-operated mice.

To further confirm that LTB_4_ and PAF have a cooperative role during I/R injury, we used a different model of I/R, a murine model of myocardial ischaemia. PAFR^−/−^ and control littermate mice underwent a 30-minute occlusion period of the LAD coronary artery and 48 hrs of reperfusion, as outlined in Figure 4A. Myocardial I/R was associated with a mean IA of 31 ± 4.0% in vehicle-treated mice (Fig. 4B). The IA was reduced by 55% (*P* < 0.01) when mice were pre-treated with the LTB_4_ receptor antagonist BIIL 2084, whereas the PAF receptor antagonist SR 27417 administered alone had no effect (Fig. 4B). Yet, the concomitant administration of LTB_4_ and PAF receptor antagonists resulted in a synergistic cardioprotective effect, with an 81% reduction of IA (*P* < 0.001; Fig. 4B). In addition, plasma ROS, as assessed by blood chemiluminescence following OpZ stimulation, was increased by ∼twofold (*P* < 0.05) in vehicle-treated mice subjected to myocardial I/R compared with sham-operated mice (Fig. 4C). Although BIIL 284 and SR 27417, administered as single drugs, did not have an effect, the concomitant administration of both drugs reduced plasma ROS by 35% (*P* < 0.05; Fig. 4C). In addition, whereas myocardial Nox1 and p67phox mRNA levels were decreased, neither superoxide dismutase-1 and -2 (Sod-1, -2), catalase (Cat) nor glutathione peroxidase-1 (Gpx-1) was modulated (Fig. 4D). Along with reduced ROS, PMN infiltration was reduced following dual treatment, in particular, at the peri-infarct area, as shown in representative photomicrographs of mice pre-treated with vehicle or BIIL 284 and SR 27417 (Fig. 4E). In agreement with a protective role of lipid mediators, PAFR^−/−^ mice were partially protected from myocardial I/R injury, as shown by an IA of 19 ± 2.7% in these mice. Pre-treatment of the PAFR^−/−^ mice with BIIL 284 slightly, but not significantly, reduced IA and blood chemiluminescence following myocardial I/R (Fig. 4F).

## Discussion

In the present study, we show a pivotal role for LTs and PAF acting in a cooperative manner to regulate leucocyte trafficking, plasma extravasation and tissue injury in diverse acute inflammatory settings and species. Lipid mediators have long been known as major inflammatory mediators released by activated leucocytes 29,30 and the recognition of their role in co-ordinating the inflammatory response in a concerted manner has slowly emerged 13–34. In this regard, we have previously reported that dual blockade of both PAF and LTB_4_ receptors elicited a significant reduction in lung PMN recruitment in a rat model of pulmonary inflammation, in contrast to either drug alone 35. Here, we provide further evidence that targeting both LTB_4_ and PAF provides a much stronger anti-inflammatory effect in rabbit skeletal muscle I/R and murine myocardial I/R injury models. Through the use of an *in vivo* bioassay model that allows testing of a variety of biologically active inflammatory mediators in the same animal, our data also improve our understanding of the important role of *de novo* generation of PAF and LTs, including CysLTs, in regulating PMN migration and oedema formation in acute inflammation.

In addition to oxidative stress 36, remote lung leucosequestration, oedema and tissue injury in earlier studies of lower torso I/R in rabbits 9–37, mice/rats 38 and sheep 39 have been associated with rise in local and/or circulating levels of LTB_4_. A role for PAF, in addition to LTB_4_, in I/R-intestinal injury was also supported by pharmacological studies showing that either LTB_4_ and/or PAF receptor antagonist administration or vessel superfusion strikingly reduced PMN adhesion, infiltration and microvascular permeability alteration at early time-points following reperfusion of the superior mesenteric artery 40–41, an effect further enhanced in the diabetic state 42.

Although a definite additive effect of the two drugs could not be observed in a previous study, the results suggested that both mediators could play a partially independent role in mediating leucocyte emigration and vascular leakage 40. It may be that the optimal drug levels were not achieved to demonstrate a cooperative effect. In our studies, we performed dose-inhibition curves for the LTB_4_ and PAF antagonists against LTB_4_- and PAF-elicited PMN accumulation in the skin (data not shown). Doses of antagonists for combined drug administration in rabbits were selected as 0.01 mg/kg for BIIL 284 and 10 mg/kg for WEB 2086, which partially reduced LTB_4_- and PAF-elicited PMN accumulation by ∼45%, within the range of reported ED_50_ values for oral administration of WEB 2086 43 and below the dose of BIIL 284 necessary for full blockade of LTB_4_ receptors in rodents 44. Using this approach, we showed, in a complex pathological model such as I/R of hind limbs known to involve a variety of mediators, including cytokines, lipid mediators, complement anaphylatoxins, cell adhesion molecules and oxidative stress 45, that treatment with a single drug antagonist had at most modest beneficial effect, whereas pre-treatment with both PAF and LTB_4_ antagonists exerted a significant attenuation of I/R-elicited remote injury and blood generation of ROS (Fig. 1). The skin bioassay model allowed the investigation of the effect associated with dual drug administration in response to chemically unrelated inflammatory mediators. Indeed, our results show that PMN recruitment elicited by soluble agonists such as IL-8, TNF-α and ZAP was partially reduced in animals treated with a single drug antagonist, although with additive effect after dual PAF and LTB_4_ receptor blockade (Fig. 2). The effect of combined drug administration was less effective in reducing oedema response to locally injected CysLTs (Fig. 2). Yet, adding a CysLT1 receptor antagonist to the drug regimen also appeared to be beneficial (Fig. 2). We further examined the selectivity of the drugs towards their respective agonist using an *in vitro* chemotactic assay using isolated rabbit PMNs. As shown in Figure 3, WEB 2086 and BIIL 284 induced dose-dependent inhibition of PAF and LTB_4_, respectively, without modulating the effect of unrelated agonists. These results are in agreement with the results showing that isolated rabbit PMNs were able to synthesize LTB_4_ in response to various agonists, in particular C5a (Fig. 3). This is also consistent with our previous studies showing that chemoattractants such as C5a and fMLP induce a rapid, transient increase in immunoreactive LTB_4_ following their i.d. injection 13. In addition, C5a des-arginine 46 and IL-8 47 were shown to elicit PAF release in isolated human PMNs. Platelet-activating factor itself was reported to increase LTB_4_ through stimulating 5-lipoxygenase activity and increased arachidonic acid availability 21,22. Taken together, our results suggest that skeletal muscle I/R, potentially *via* ROS formation and elevation in soluble agonists such as C5a, promotes PAF and LTB_4_ generation, which serves to amplify the inflammatory response in a highly cooperative manner.

We further explored the effect of PAF and LTB_4_ receptor antagonists in a model of transient myocardial ischaemia in mice. In agreement with a deleterious effect of elevated PAF concentrations 49 and a potentially harmful effect of arachidonic acid metabolites 50 in myocardial I/R, we observed a potent cardioprotective effect of LTB_4_ receptor antagonist pre-treatment. Surprisingly, the administration of the PAF receptor antagonist did not reduce myocardial infarct area. Additional studies are needed to address this point. However, a synergistic effect in reducing infarct area was observed with the concomitant administration of PAF and LTB_4_ receptor antagonists (Fig. 4B), and, in a similar manner, only dual drug administration reduced OpZ-stimulated blood ROS formation in mice subjected to myocardial I/R (Fig. 4C), demonstrating that a cross-talk between the two lipid mediators is important in this pathophysiological context. Interestingly, myocardial Nox1, which is involved in ROS generation 51, is decreased in animals pre-treated with both receptor antagonists, along with p67phox, the activator sub-unit of Nox2 52. However, none of the anti-oxidant enzymes tested, including Sod-1 and -2, catalase and Gpx-1, were modulated at the gene level (Fig. 4D). In accordance with an important role for PAF in myocardial I/R injury, we observed a comparatively smaller infarct size in mice deficient in PAF receptor. This is in agreement with an important role of PAF in myocardial I/R 53. Yet, pre-treatment of the PAFR^−/−^ mice with the same, sub-maximal dose of BIIL 284 as that administered in wild-type mice did not show significantly greater reduction in the myocardial infarct zone in that model (Fig. 4F). Altogether, our results support a cooperative role for LTB_4_ and PAF in reducing infarct area.

In conclusion, the present study shows a cooperative behaviour between LTB_4_ and PAF, and, to a lesser degree, CysLT, in promoting LTB_4_, PAF and chemically unrelated soluble agonists to induce PMN recruitment, oedema and tissue injury following I/R. This is reinforced by the fact that the partial inhibition observed with either antagonist alone was enhanced by the concomitant administration of receptor antagonists. To extend these observations, the potentially cooperative effects of lipid mediator in regulating PMN trafficking were investigated over a range of different species, tissues and acute inflammatory conditions. Altogether, these studies support targeting the action of more than one lipid mediator in I/R to ensure maximal therapeutic impact.
